# Pulmonary pulse wave velocity as assessed with velocity-encoded MRI

**DOI:** 10.1186/1532-429X-14-S1-P280

**Published:** 2012-02-01

**Authors:** Heynric Grotenhuis, Wouter Stomp, Jos J Westenberg, Albert de Roos

**Affiliations:** 1 Radiology, Leiden University Medical Center, Leiden, Netherlands

## Summary

Pulmonary pulse wave velocity has proven to be an excellent indicator of pulmonary vascular wall stiffness. In this study a novel MRI method was evaluated, showing rapid assessment of pulmonary pulse wave velocity with excellent repeatability and observer reproducibility.

## Background

Pulmonary pulse wave velocity has proven to be an excellent indicator of pulmonary vascular wall stiffness. The main objective of this study was to evaluate a novel magnetic resonance imaging (MRI) method to rapidly assess pulmonary pulse wave velocity (PWV) and to test repeatability and observer reproducibility of the proposed technique.

## Methods

In 15 healthy volunteers (7 male; mean±SD age 19±6 years) pulmonary PWV was prospectively assessed between the proximal main pulmonary artery (MPA) and just before the first branching of the distal left pulmonary artery (LPA). One-directional through-plane velocity-encoded (VE) MRI using breath holds was used to assess the pulmonary flow at the levels of MPA and distal LPA with a temporal resolution of 15 ms. Pulmonary PWV was calculated by dividing the pulmonary path length between the measurement sites by the transit time between the arrival of the systolic wave front at these sites. The volunteers were studied twice to test reproducibility in PWV-assessment.

## Results

Results are expressed as mean±SD, Pearson correlation coefficient (PCC) and intraclass correlation (ICC). Acquisition time of the MRI examination was 2±1 minutes and image analysis lasted for 2±1 minutes. Both observers showed good agreement for pulmonary distance (observer 1: 52.2±11.0 mm vs. observer 2: 52.7±11.1 mm; PCC=0.99) and pulmonary PWV (observer 1: PWV 2.73±1.05 m/s vs. observer 2: 2.76±1.06 m/s; PCC=0.99). Reproducibility of pulmonary PWV was high for both observers (observer 1: 2.73±1.05 m/s vs. 2.72±1.04 m/s, ICC=1.00, p<0.01; observer 2: 2.76±1.06 m/s vs. 2.75±1.06 m/s, ICC=1.00, p<0.01; ICC observer 1 vs. observer 2: 0.99, p<0.01).

## Conclusions

Rapid non-invasive VE MRI assessment of pulmonary pulse wave velocity shows low interobserver and intraobserver variability and can be determined with high reproducibility.

## Funding

No disclosures.

